# Nutrition Knowledge and Low Energy Availability Risk in Ladies Gaelic Football Players

**DOI:** 10.3390/nu18152413

**Published:** 2026-07-24

**Authors:** Jennifer M. Connolly, Andrea M. McNeilly

**Affiliations:** 1 Sports and Exercise Sciences Research Institute, Ulster University, Coleraine BT52 1SA, Northern Ireland, UK; 2 Department of Medical Foundations, Ross University School of Medicine, Two Mile Hill, St. Michael BB11093, Barbados

**Keywords:** low energy availability, RED-S, sport nutrition knowledge, female team sport athletes, Ladies Gaelic Football, LEAF-Q, athlete nutrition, menstrual health

## Abstract

Background: Nutrition knowledge (NK) may influence dietary behaviours and athlete health; however, limited research has examined the relationship between NK and risk of low energy availability (LEA) in female team sport athletes. Objective: This study aimed to assess NK and LEA risk in female Ladies Gaelic Football (LGF) players and to examine the relationship between these variables. Results: Female LGF players (*n* = 58) aged 18–35 years completed a 75-item online questionnaire comprising the Abridged Nutrition for Sport Knowledge Questionnaire (ANSKQ) and the Low Energy Availability in Females Questionnaire (LEAF-Q). Mean total ANSKQ score was 53.1 ± 7.6%, categorized as “average”, while the mean sport nutrition knowledge (SNK) score was 48.2 ± 8.5%, categorized as “poor”. Lower micronutrient knowledge scores were observed among younger players and those with lower educational attainment (*p* < 0.05), while players with prior nutrition education demonstrated greater supplement-related knowledge (*p* < 0.05). Overall, 56.9% of the participants were classified as being at risk of LEA according to LEAF-Q criteria. LEA risk differed according to playing level and nutrition education status (*p* < 0.05); however, no association between overall NK and LEA risk was identified. Players identified as being at risk of LEA also demonstrated greater menstrual dysfunction and injury-related absence. Conclusions: These findings indicate a high prevalence of LEA risk and suboptimal sport NK among female LGF players and suggest that NK alone may be insufficient to protect against LEA risk in female team sport athletes. Ongoing screening, nutrition education, and broader athlete support strategies are therefore warranted in this population.

## 1. Introduction

Optimal nutritional strategies are important for supporting athletic performance, recovery, and overall athlete health [1]. Despite growing awareness of sports nutrition, many athletes fail to meet current nutritional recommendations [2]. Female athletes may be particularly vulnerable to inadequate energy and nutrient intake, including inadequate iron, vitamin D, and calcium intake [3]. These inadequacies may negatively impact performance and contribute to health consequences, including menstrual dysfunction, impaired bone health, increased injury risk, and illness [4]. Collectively, these physiological consequences are described within the framework of Relative Energy Deficiency in Sport (RED-S), for which low energy availability (LEA) is considered the primary underpinning factor [4].

Energy availability (EA) is defined as the amount of dietary energy remaining to support physiological function after exercise energy expenditure has been subtracted from energy intake, relative to fat-free mass (FFM) [5]. LEA occurs when insufficient energy remains to adequately support normal physiological processes and athlete health [4]. Despite emerging evidence indicating individual variation in response to LEA, it is commonly defined as <30 kcal/kg FFM/day [6]. Direct assessment of EA can be methodologically challenging in free-living athletes because accurate measurement of both dietary intake and exercise energy expenditure is required [7]. Consequently, screening tools such as the Low Energy Availability in Females Questionnaire (LEAF-Q) are commonly used to identify female athletes at risk of LEA [8]. Recent studies have identified a high prevalence of LEA risk across female team sports, including Australian football, soccer, rugby, and Gaelic games athletes [9,10,11,12,13,14].

Nutrition knowledge (NK) is considered an important and potentially modifiable determinant of dietary behaviour in athletes [15]. While greater NK has been associated with healthier dietary patterns and improved dietary intake in some athlete populations [2,16,17], emerging evidence suggests that NK alone may not consistently translate into optimal nutritional practices or reduced LEA risk [18]. Previous research also suggests that NK may be influenced by educational attainment, previous nutrition education, and athletic level, although findings remain inconsistent across sporting populations [19,20,21]. Understanding how NK differs according to these athlete characteristics may help identify athletes who would benefit most from targeted nutrition education interventions [21]. Furthermore, the amateur nature of field-based team sports such as Gaelic football presents additional challenges to accessing qualified nutrition support, with potential implications for NK and dietary behaviours [21]. However, inadequate energy intake is multifactorial, with behavioural, environmental, and psychosocial factors also contributing to LEA risk. Consequently, the relationship between NK and LEA risk remains unclear, particularly in female team sport athletes.

Ladies Gaelic Football (LGF) is the most popular competitive female sport in Ireland [22] and has been recognized as one of the fastest-growing female sports in Europe [23]. Research interest in LGF has increased in recent years, with studies examining anthropometry, performance characteristics, injury risk and training demands [24,25,26,27]. Existing studies have reported suboptimal NK, inadequate macro- and micronutrient intake and elevated LEA risk in female Gaelic games athletes [14,20,21,28,29,30,31]. However, despite increasing recognition of LEA risk in female team sport athletes, limited research has specifically examined the relationship between NK and LEA risk in LGF players. Therefore, the aim of this study was to assess the relationship between NK and the risk of LEA in female LGF players. We hypothesized that players would demonstrate suboptimal nutrition knowledge and a substantial prevalence of LEA risk and that lower nutrition knowledge would be associated with greater LEA risk.

## 2. Methods

### 2.1. Participants

**Figure 1:**
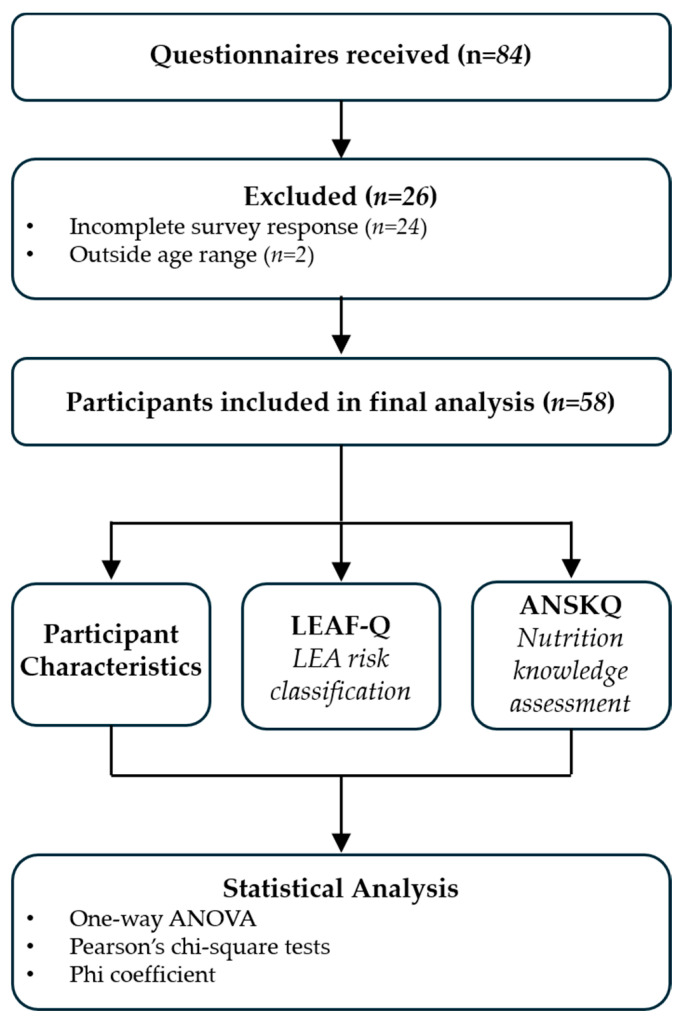
Study flow diagram illustrating participant recruitment, exclusions, data collection, and statistical analyses. ANSKQ: Abridged Nutrition for Sport Knowledge Questionnaire; LEAF-Q: Low Energy Availability in Females Questionnaire; LEA: low energy availability; ANOVA: analysis of variance.

LGF players (*n* = 58) were recruited to participate in this cross-sectional observational study. Participants were recruited by email and social media and screened prior to participation to ensure they met study inclusion criteria: healthy female Gaelic football players aged 18–35 years who trained at least twice per week and competed at either club level (non-elite) or county level (elite), reflecting the two primary competitive standards within Gaelic football. Participants were excluded if they were pregnant, breastfeeding, or postmenopausal, or if they had any chronic illness which could influence dietary intake or nutrient absorption. The study was approved by Ulster University’s Research Ethics Committee (UUREC; SESRI-24-001-A). All participants consented to participate in the study. The study design, participant recruitment, exclusions, data collection and statistical analysis are summarized in Figure 1. 

### 2.2. Instruments

Participants were required to complete an anonymous online questionnaire comprising 75 questions divided into three sections. Section one included 15 questions relating to participant demographics (age, weight, height, education, training, and sport). Participants were also asked whether they had any formal education or training in nutrition, dietetics, or another health-related profession and, where applicable, were invited to provide additional details. Responses were subsequently categorized as yes/no for analysis. Section two included 35 questions from the validated Abridged Nutrition for Sport Knowledge Questionnaire (ANSKQ) relating to NK [15,32]. The questionnaire comprises 11 general nutrition knowledge (GNK) items and 24 sport nutrition knowledge (SNK) items, covering key nutrition topics including weight management, macronutrients, micronutrients, hydration, dietary supplements, sport nutrition, and alcohol [32]. ANSKQ scoring allocated one point for each correct response, while incorrect or ‘unsure’ responses received a score of zero. Correct responses were summed to generate a total NK score (range 0–35), a general NK score (range 0–11), and a sport NK score (range 0–24). NK was categorized as poor (0–49%), average (50–64%), good (65–74%), and excellent (≥75%) [15,32]. Section three included 25 questions assessing physiological symptoms associated with LEA using the validated Low Energy Availability in Females Questionnaire (LEAF-Q) [8]. The LEAF-Q assesses three domains: injury history, gastrointestinal function, and menstrual function. Responses were scored according to the validated LEAF-Q scoring system, with individual items assigned weighted scores based on symptom severity. Total LEAF-Q scores were summed, and participants with a score of ≥8 were classified as being at risk of LEA [8]. Study data were collected and managed using Research Electronic Data Capture (REDCap) electronic data capture tools (Vanderbilt University, Nashville, TN, USA) hosted by Ulster University [33].

### 2.3. Statistical Analysis

Statistical analyses were performed using SPSS version 27.0 (IBM Corp., Armonk, NY, USA). Normality of the data was assessed using the Shapiro–Wilk test. Categorical data are expressed as absolute numbers and as percentages (%). Continuous data are expressed as mean ± standard deviation (SD). Continuous data that were not normally distributed are expressed as median values (IQR). Independent-sample *t*-tests and one-way analysis of variance (ANOVA) were used, where appropriate, to assess differences in NK and LEAF-Q scores across age, BMI, playing level, education level, nutrition education status, and training volume. Chi-square tests were used to examine associations between categorical data, and Pearson correlation coefficients were used to assess relationships between continuous variables. Correlation coefficients were interpreted as follows: very weak (<0.20), weak (0.20–0.39), moderate (0.40–0.59), strong (0.60–0.79), and very strong (>0.80). Effect sizes for chi-square analyses were interpreted as small (w = 0.10), medium (w = 0.30), and large (w = 0.50). Significance was set at *p* < 0.05.

## 3. Results

### 3.1. Participant Characteristics

Participant characteristics are presented in Table 1. Participants had a mean age of 24.6 ± 5.6 years and a mean BMI of 23.9 ± 4.5 kg/m^2^. Most participants had completed undergraduate or postgraduate education (62.1%), had no previous nutrition education (72.5%), and competed at the club level (70.6%). Overall, 56.9% (*n* = 33) of participants were classified as being at risk of LEA (LEAF-Q ≥ 8). Chi-square analysis demonstrated significant associations between LEA classification and previous nutrition education (*p* < 0.001) and between LEA classification and playing calibre (*p* < 0.05).

### 3.2. Nutrition Knowledge (NK)

NK scores for the overall cohort and demographic subgroups are presented in Table 2. Overall, the mean ANSKQ score was categorized as “average” (53.1 ± 7.6%), with mean general nutrition knowledge (GNK) scores higher than sports nutrition knowledge (SNK) scores (63.3 ± 16.2% vs. 48.2 ± 8.5%). No significant differences were observed in overall ANSKQ, GNK, or SNK scores across demographic subgroups (Table 2). Analysis of ANSKQ subsection scores demonstrated that participants aged 24 years or younger had significantly lower micronutrient knowledge scores than older participants (*p* < 0.05). Similarly, participants whose highest level of education was secondary school demonstrated significantly lower micronutrient knowledge scores than those with tertiary education (*p* < 0.05). Participants with prior nutrition education also demonstrated significantly greater supplement knowledge scores than those without prior nutrition education (*p* < 0.05).

### 3.3. LEAF-Q Scores and Participant Characteristics

Overall, 56.9% (*n* = 33) of participants were classified as being at risk of LEA (LEAF-Q ≥ 8), while 43.1% (*n* = 25) were classified as not at risk (LEAF-Q < 8).

Comparisons between participants classified as at risk and not at risk of LEA are presented in Table 3. Mean LEAF-Q score was significantly higher in the at-risk group than in the no-risk group (12.9 ± 3.7 vs. 5.0 ± 2.1, *p* < 0.001). No significant differences were observed between groups for age, height, weight, BMI, weekly training volume, general nutrition knowledge (GNK), sport nutrition knowledge (SNK), or overall nutrition knowledge (ANSKQ) scores (all *p* > 0.05).

Analysis of categorical variables demonstrated no significant association between LEA risk and education level (χ^2^(2) = 0.73, *p* = 0.695). However, significant associations were observed between LEA risk and both prior nutrition education (χ^2^(1) = 16.74, *p* < 0.001, Φ = 0.537) and playing calibre (χ^2^(1) = 4.58, *p* = 0.032, Φ = 0.281). Among participants classified as at risk of LEA, 48.5% reported prior nutrition education, whereas none of the participants classified as not at risk reported prior nutrition education. In addition, a greater proportion of participants classified as at risk competed at a non-elite level compared with those classified as not at risk (81.8% vs. 56.0%).

### 3.4. LEAF-Q Questions

Participants classified as being at risk of LEA reported significantly greater injury-related absence from training or competition compared with the no-risk group (72.7% vs. 32.0%, *p* < 0.05), with longer injury-related absences more common among at-risk participants. Menstrual dysfunction was also more prevalent among participants at risk of LEA. Compared with the no-risk group, at-risk participants reported higher rates of abnormal menstruation, altered bleeding duration, fewer menstrual cycles over the previous 12 months, prolonged menstrual absence and menstrual changes associated with increased exercise frequency, intensity, or duration (*p* < 0.05 for all comparisons). Detailed LEAF-Q responses are presented in Supplementary Table S1.

## 4. Discussion

The aims of this study were to assess nutrition knowledge (NK) and risk of low energy availability (LEA) in female LGF players, to explore demographic characteristics associated with NK scores and LEA risk, and to examine the relationship between NK and LEA risk. The main findings of this study were that participants demonstrated “average” overall nutrition knowledge, “poor” sport nutrition knowledge, and a high prevalence of LEA risk, with 56.9% of participants classified as being at risk according to LEAF-Q criteria [8]. Despite suboptimal NK and substantial LEA risk prevalence within the cohort, no association between NK and LEA risk was identified, suggesting that factors beyond NK alone may contribute to LEA risk in female team sport athletes. Younger participants and those with secondary-level education demonstrated lower micronutrient knowledge scores, while LEA risk differed according to playing level and nutrition education status.

A substantial proportion of participants in the current study were identified as being at risk of LEA (56.9%). This prevalence is consistent with previous reports in female team sport athletes, including soccer, rugby, Australian football, and Gaelic games players [9,10,11,12,14]. The findings therefore add to growing evidence that LEA risk may be common among female team sport athletes, despite increasing awareness of RED-S and athlete nutritional intake. Given the physiological and performance consequences associated with LEA and RED-S, including menstrual dysfunction, impaired bone health, increased injury risk, and reduced recovery capacity, these findings highlight the importance of ongoing screening and monitoring practices within female Gaelic games athletes.

Participants in the current study demonstrated “average” overall NK, although sport nutrition knowledge scores were categorized as “poor” and were lower than general nutrition knowledge scores. These findings are consistent with previous studies in female Gaelic games athletes and other athlete populations reporting suboptimal sport-specific nutrition knowledge despite reasonable awareness of general healthy eating principles [15,20,21]. Lower micronutrient knowledge scores observed among younger participants and those with lower educational attainment may reflect reduced exposure to formal nutrition education or less experience managing training and recovery demands. The disparity observed between GNK and SNK scores may suggest that athletes are more familiar with general dietary recommendations than with sport-specific nutritional strategies relating to recovery, fuelling, supplementation, and performance nutrition.

Although the overall NK of participants was classified as “average”, SNK remained “poor”. The amateur nature of LGF may contribute to these findings, as athletes may have inconsistent access to qualified sport nutrition practitioners while balancing training alongside employment or education. Consequently, opportunities for structured nutrition education and ongoing nutritional support may be limited [20,21]. Previous research has similarly suggested that educational attainment, previous nutrition education, and access to nutrition support may influence athletes’ NK and contribute to the variability observed across amateur sporting populations [19,20,21].

Despite suboptimal NK and a high prevalence of LEA risk within the cohort, no association between overall NK and LEA risk was identified. Similarly, Pai and colleagues reported high rates of LEA risk and poor NK among female team sport athletes, yet found no significant differences in NK between athletes classified as at risk and not at risk of LEA [18]. Earlier work by Magee et al. reported lower NK scores among collegiate soccer athletes identified as being at risk of LEA; however, the study involved a relatively small cohort. Interestingly, participants with prior nutrition education demonstrated significantly greater supplement knowledge; however, this did not translate into a lower risk of LEA [10]. While this finding appears contradictory to previous reports linking greater NK with improved dietary practices, understanding nutritional requirements for athletic performance does not necessarily translate into optimal dietary behaviours [32,34]. The complexity underlying LEA is likely multifactorial and may involve both intentional and unintentional under-fuelling alongside behavioural, psychosocial, environmental, and sport-specific influences [34,35]. Practical barriers may include time constraints, financial limitations, lack of motivation, body image pressures, and disordered eating behaviours, all of which may also contribute to inadequate energy intake in athletes [35,36]. Together, these findings suggest that NK alone may not adequately protect against LEA risk in female athletes and highlight the potential limitations of knowledge-based approaches alone.

Players identified as being at risk of LEA demonstrated significantly greater menstrual dysfunction and injury-related absence from training and competition compared with players not at risk of LEA. Menstrual disturbances, including abnormal menstruation, reduced menstrual frequency, prolonged menstrual absence, and exercise-associated menstrual changes, are recognized markers of LEA, commonly described within the RED-S framework [4]. These findings are concerning given the potential implications of chronic LEA for reproductive health, bone health, injury risk, recovery, and athletic performance [4]. In the current study, athletes at risk of LEA were significantly more likely to report abnormal menstruation, fewer menstrual cycles during the preceding year, a history of menstrual cessation lasting three months or longer, and menstrual disturbances associated with increased exercise participation. Moderate-to-large effect sizes were observed across these variables, suggesting meaningful associations between LEA risk and menstrual dysfunction within this cohort. These findings are consistent with previous literature identifying menstrual dysfunction as one of the most common and clinically relevant manifestations of prolonged energy deficiency in female athletes [4,37]. However, menstrual dysfunction is multifactorial and should not be attributed solely to LEA. Training load, exercise stress, psychological stress, hormonal contraceptive use, and underlying medical or endocrine conditions may also influence menstrual function [4]. Consequently, the menstrual disturbances observed in the present cohort are likely to reflect a combination of low energy availability and other physiological and training-related factors.

The increased injury-related absence observed among athletes at risk of LEA further supports the broader pattern of RED-S-related health consequences identified within this cohort. Previous studies have linked LEA and RED-S with impaired recovery, increased susceptibility to illness, reduced training adaptation, and a greater risk of musculoskeletal injury in female athletes [4,37]. Athletes experiencing prolonged periods of inadequate energy availability may lack sufficient energy to support both physiological function and the demands of training, potentially compromising recovery and increasing vulnerability to injury. Collectively, these findings suggest that the elevated LEA risk observed within this cohort is associated with several clinically relevant outcomes that may adversely affect both athlete health and performance.

Given the high prevalence of LEA risk identified in the current cohort, ongoing screening and educational initiatives targeting RED-S awareness, menstrual health, recovery, and appropriate fuelling practices may be beneficial for female LGF players and support staff [4]. Preventive educational programs and screening strategies aimed at identifying athletes at risk of LEA and RED-S have previously been recommended to support early intervention and reduce the risk of long-term health consequences [38]. Previous intervention studies have further demonstrated that nutrition education programs can improve sport nutrition knowledge, dietary intake, and nutrition-related behaviours in athletes [39,40,41]. More recently, Fahrenholtz et al. reported improvements in sport nutrition knowledge, dietary intake, sports nutrition habits, and eating behaviours following a 16-week intervention involving online lectures and weekly counselling sessions in Irish female endurance athletes identified as being at risk of RED-S [42]. Educational workshops have also been shown to improve NK and willingness to adopt healthier dietary practices in adolescent Gaelic games athletes [43].

While nutrition education remains an important component of athlete support, the absence of an association between NK and LEA risk in the present study suggests that improving knowledge alone may be insufficient to ensure adequate energy availability in female LGF players. Translating nutrition knowledge into consistent dietary practice may require ongoing reinforcement through practical fuelling advice, early identification of athletes at risk of LEA, and continued support from qualified practitioners [4,44]. Given the amateur nature of LGF, where access to sports nutrition professionals may be limited [28,30], coaches and other support staff are well positioned to reinforce evidence-based nutrition messages and facilitate appropriate referral to qualified practitioners when concerns regarding LEA or RED-S arise [44]. Strengthening collaboration between coaches and qualified sports nutrition practitioners may therefore represent a practical strategy to ensure that athletes receive consistent, evidence-based nutrition messages while improving athlete support within amateur LGF settings [19,44].

The current study has several strengths. To the authors’ knowledge, this is one of the few studies to simultaneously examine NK and LEA risk in female Gaelic games athletes using validated assessment tools. The study also contributes to the limited body of nutrition-related research in LGF players and provides novel insight into the relationship between NK and LEA risk in this population.

Several limitations should be acknowledged. The cross-sectional study design limits the ability to infer causality between NK and LEA risk. In addition, LEA risk was assessed using LEAF-Q, which is a screening tool rather than a diagnostic measure of LEA or RED-S. The LEAF-Q was originally validated in endurance athletes, and its application in team sport populations may have limitations [8]. All data were self-reported and may therefore be subject to recall bias and reporting inaccuracies. Menstrual function data may also have been influenced by symptom normalization, underreporting, or hormonal contraceptive use. Direct assessments of dietary intake, exercise energy expenditure, and objective physiological markers of LEA were also not included within the current study design. Although playing calibre (elite vs. non-elite) was assessed, all participants were amateur LGF players, and playing calibre should not be interpreted as a measure of competitive experience. The study also did not assess playing experience, competitive success, access to qualified nutrition support, or the nutrition knowledge of coaches and support staff. Consequently, the potential influence of these factors on NK and LEA risk could not be evaluated and warrants further investigation. Finally, the relatively modest sample size may limit the generalizability of the findings to the wider LGF population.

## 5. Conclusions

Results from the current study indicate a high prevalence of LEA risk and suboptimal sport nutrition knowledge in female LGF players. Although an association between NK and LEA risk was not identified, the findings suggest that NK alone may be insufficient to protect against LEA risk in female team sport athletes. Given the potential long-term health and performance consequences associated with LEA and RED-S, ongoing screening, education, and broader multidisciplinary support strategies warrant consideration within this population. Future research should investigate the complex factors influencing LEA risk and evaluate targeted interventions designed to support athlete health, nutritional practices, and performance in female Gaelic games athletes.

## Figures and Tables

**Table 1:** Participant characteristics of female Gaelic football players according to LEAF-Q classification.

Characteristic		All Participants (*n* = 58)	LEAF-Q < 8 (*n* = 25)	LEAF-Q ≥ 8 (*n* = 33)
Age (years)		24.6 ± 5.6	24.4 ± 6.2	24.8 ± 5.2
Height (cm)		166.9 ± 6.60	165.1 ± 6.0	168.3 ± 6.8
Weight (kg)		66.43 ± 12.3	65.4 ± 11.4	67.2 ± 13.1
BMI (kg/m^2^)		23.9 ± 4.5	24.1 ± 4.8	23.7 ± 4.3
Highest level of education				
	Secondary School	22 (37.9%)	11 (44.0%)	11 (33.3%)
	Undergraduate	25 (43.1%)	10 (40.0%)	15 (45.5%)
	Postgraduate	11 (19.0%)	4 (16%)	7 (21.2%)
Nutrition education				
	Yes	16 (27.5%)	0 (0.0%)	16 (45.7%)
	No	42 (72.5%)	25 (100%)	17 (51.5%)
Playing calibre				
	Non-elite	41 (70.6%)	14 (56.0%)	27 (81.8%)
	Elite	17 (29.3%)	11 (44.0%)	6 (18.8%)
Training volume (h/week)				
	<10	33 (55.9%)	13 (52%)	20 (60%)
	≥10	25 (43.1%)	12 (48%)	13 (40%)
ANSKQ Rating				
	Poor	17 (29.3%)	9 (36.0%)	8 (24.2%)
	Average	31 (62.1%)	10 (40.0%)	21 (63.6%)
	Good	10 (8.6%)	6 (24.0%)	4 (12.1%)
	Excellent	0 (0.0%)	0 (0.0%)	0 (0.0%)

Values are presented as mean ± standard deviation (SD) or *n* (%). LEAF-Q: Low Energy Availability in Females Questionnaire; BMI: body mass index; cm: centimetres; kg: kilograms; h: hour; NK: nutrition knowledge; ANSKQ: Abridged Nutrition for Sport Knowledge Questionnaire.

**Table 2:** Nutrition knowledge scores for the total sample and demographic subgroups.

Group		(*n*)	ANSKQ (%)	GNK (%)	SNK (%)
Total sample		58	53.1 ± 7.6	63.3 ± 16.2	48.2 ± 8.5
Age (years)					
	18–24	34	52.7 ± 8.6	65.5 ± 17.3	46.5 ± 8.5
	25–30	14	51.9 ± 6.5	59.1 ± 15.4	48.4 ± 8.3
	>30	10	56.2 ± 4.9	61.8 ±13.4	53.5 ± 6.8
BMI (kg/m^2^)					
	<18	2	51.5 ± 10.4	59.1 ± 6.4	47.8 ± 12.3
	18–24.9	37	52.4 ± 7.9	60.1 ± 15.7	48.1 ± 8.1
	>25	19	53.1 ± 7.6	68.8 ± 16.9	47.8 ± 9.4
Education level					
	Secondary School	22	53.5 ± 7.5	65.7 ± 15.3	47.6 ± 9.3
	Undergraduate	25	53.2 ± 7.2	62.9 ± 18.5	48.5 ± 6.5
	Postgraduate	11	52.1 ± 9.6	59.5 ± 12.4	48.6 ± 11.1
Nutrition education					
	Yes	16	55.5 ± 8.8	68.2 ± 19.1	49.5 ± 8.2
	No	42	52.2 ± 7.0	61.5 ± 14.9	47.7 ± 8.6
Playing calibre					
	Elite	17	50.2 ± 8.8	61.0 ± 17.8	45.0 ± 9.7
	Non-elite	41	54.3 ± 6.9	64.3 ± 15.7	49.5 ± 7.6
Training volume (h/week)					
	<10	33	52.5 ± 7.2	62.2 ± 15.4	47.8 ± 8.7
	≥10	25	53.9 ± 8.3	64.7 ± 17.5	48.7 ± 8.2
At risk of LEA					
	Yes	33	54.4 ± 7.7	65.6 ± 16.4	49.0 ± 7.7
	No	25	51.5 ± 7.4	60.4 ± 15.9	47.1 ± 9.4

Nutrition knowledge scores are presented as mean ± standard deviation (SD). ANSKQ: Abridged Nutrition for Sport Knowledge Questionnaire; GNK: general nutrition knowledge; SNK: sport nutrition knowledge; BMI: body mass index; LEA: low energy availability; h: hour.

**Table 3:** Comparison of continuous (Panel **A**) and categorical (Panel **B**) participant characteristics between participants classified as not at risk (LEAF-Q < 8) and at risk (LEAF-Q ≥ 8) of low energy availability (LEA).

**(A) Continuous Variables**				
**Variable**		**LEAF-Q < 8 (*n* = 25)**	**LEAF-Q** ≥**8 (*n* = 33)**	***p*** **-Value**
LEAF-Q score		5.0 ± 2.1	12.9 ± 3.7	<0.001
Age (years)		24.5 ± 6.1	24.8 ± 5.2	0.841
Height (cm)		165.1 ± 6.0	168.3 ± 6.8	0.071
Weight (kg)		65.4 ± 11.4	67.2 ± 13.1	0.579
BMI (kg/m^2^)		24.1 ± 4.8	23.7 ± 4.3	0.731
Training volume (h/week)		9.6 ± 3.1	9.9 ± 3.9	0.796
GNK (%)		60.3 ± 15.9	65.6 ± 16.3	0.229
SNK (%)		47.1 ± 9.5	49.0 ± 7.7	0.403
ANSKQ (%)		51.4 ± 7.4	54.4 ± 7.7	0.146
**(B) Categorical Variables**				
**Variable**		**LEAF-Q < 8 (*n* = 25)**	**LEAF-Q > 8 (*n* = 33)**	***p*** **-Value**
Education level				
	Secondary	11 (44.0%)	11 (33.3%)	
	Undergraduate	10 (40.0%)	15 (45.5%)	
	Postgraduate	4 (16.0%)	7 (21.2%)	0.695
Nutrition education				
	Yes	0 (0.0%)	16 (48.5%)	
	No	25 (100.0%)	17 (51.5%)	<0.001
Playing calibre				
	Elite	11 (44.0%)	6 (18.2%)	
	Non-elite	14 (56.0%)	27 (81.8%)	0.032

Continuous variables are presented as mean ± standard deviation (SD) and were compared using one-way ANOVA. Categorical variables are presented as *n* (%) and were compared using Pearson’s chi-square or Fisher’s exact test, where appropriate. Statistical significance was accepted at *p* < 0.05. LEAF-Q: Low Energy Availability in Females Questionnaire; LEA: low energy availability; GNK: general nutrition knowledge; SNK: sport nutrition knowledge; ANSKQ: Abridged Nutrition for Sport Knowledge Questionnaire; BMI: body mass index; h: hour.

## Data Availability

The original contributions presented in this study are included in the article/Supplementary Material. Further inquiries can be directed to the corresponding author

## Supplementary Materials

The following supporting information can be downloaded at https://www.mdpi.com/article/10.3390/nu18152413/s1, Table S1: Frequency of LEAF-Q responses according to LEA risk classification.
