# Supplementary material for: Nutrition Knowledge and Low Energy Availability Risk in Ladies Gaelic Football Players
Source: Nutrients. 2026 Jul 24;18(15):2413. doi: 10.3390/nu18152413 (PMC13467711; doi:10.3390/nu18152413)
Supplement: Supplementary file 1 [file nutrients-18-02413-s001.zip › nutrients-4405466-supplementary.pdf]

Supplementary Table S1.

Frequency of LEAF-Q responses according to LEA risk classification

LEAF-Q Question	Response	All Participants *n* (%)	LEAF-Q <8 n (%)	LEAF-Q ≥8 n (%)	Φ / Cramer's V	*p*-value
**Injury history**						
**Absences from training or competition due to injury**	No, not at all	26 (44.8)	17 (68.0)	9 (27.3)	0.413	<0.05
	Yes, once or twice	28 (48.3)	7 (28.0)	21 (63.6)		
	Yes, three or four times	3 (5.2)	1 (4.0)	2 (6.1)		
	Yes, five or more times	1 (1.7)	0 (0)	1 (3.0)		
**Number of days absent from training or competition due to injury**	1–7 days	16 (48.5)	6 (24.0)	10 (30.3)	0.495	<0.01
	8–14 days	9 (27.3)	2 (8.0)	7 (21.2)		
	15–21 days	5 (15.2)	0 (0)	5 (15.1)		
	≥22 days	3 (9.1)	0 (0)	3 (9.1)		

**LEAF-Q Question**	**Response**	**All Participants *n* (%)**	**LEAF-Q <8 *n* (%)**	**LEAF-Q ≥8 *n* (%)**	**Φ / Cramer's V**	***p*-value**
**Gastrointestinal function**						
**Do you feel gaseous or bloated in the abdomen when you do not have your period?**	Several times/day	13 (22.4)	4 (16.0)	9 (27.3)	0.182	0.31
	Several times/week	16 (27.6)	4 (16.0)	12 (36.4)		
	Once/twice weekly or less	22 (37.9)	12 (48.0)	10 (30.3)		
	Rarely/never	7 (12.1)	5 (20.0)	2 (6.0)		
**Do you get cramps or stomachache which cannot be related to your menstruation?**	Several times/day	7 (12.1)	2 (8.0)	5 (15.1)	0.367	<0.05
	Several times/week	4 (6.9)	1 (4.0)	3 (9.1)		
	Once/twice weekly or less	22 (37.9)	6 (24.0)	16 (48.5)		
	Rarely/never	25 (43.1)	16 (64.0)	9 (27.3)		

LEAF-Q Question	Response	All Participants *n* (%)	LEAF-Q <8 *n* (%)	LEAF-Q ≥8 *n* (%)	Φ / Cramer's V	*p*-value
Menstrual Function						
**Do you have normal menstruation?**	Yes	40 (69.0)	23 (92.0)	17 (51.5)	0.407	<0.01
	No	18 (31.0)	2 (8.0)	16 (48.5)		
**When was your last period?**	0-4 weeks ago	47 (81.0)	24 (96.0)	23 (69.7)	NS	NS
	1-2 months ago	9 (15.5)	1 (4.0)	8 (24.2)		
	3-4 months ago	1 (1.7)	0 (0)	1 (3.0)		
	≥5 months ago	1 (1.7)	0 (0)	1 (3.0)		
**For how many days do you normally bleed?**	1-2 days	3 (5.2)	0 (0)	3 (9.1)	0.416	<0.05
	3-4 days	25 (43.1)	16 (64.0)	9 (27.3)		
	5-6 days	24 (41.4)	8 (32.0)	16 (48.5)		
	7-8 days	3 (5.2)	1 (4.0)	2 (6.1)		
	≥9 days	3 (5.2)	0 (0)	3 (9.1)		
**How many periods have you had in the last year?**	≥12	23 (39.7)	14 (56.0)	9 (27.3)	0.525	<0.01
	9-11	17 (29.3)	10 (40.0)	7 (21.2)		
	6-8	8 (13.8)	1 (4.0)	7 (21.2)		
	3-5	10 (17.2)	0 (0)	10 (30.3)		

**Have your periods ever stopped for 3 consecutive months or longer (excluding pregnancy or breastfeeding)?**	No, never	37 (63.8)	22 (88.0)	15 (45.5)	0.440	<0.001
	Yes, previously	20 (34.5)	3 (12.0)	17 (51.5)		
	Yes, currently	1 (1.7)	0 (0)	1 (3.0)		
**Do you experience menstrual changes with increased exercise intensity, frequency, or duration?**	Yes	29 (50.0)	6 (24.0)	23 (69.7)	0.453	<0.001
	No	29 (50.0)	19 (76.0)	10 (30.3)		
**If yes, how?**	I bleed less	12 (20.7)	3 (8.0)	9 (27.3)	0.477	<0.01
	I bleed more	2 (3.4)	0 (0)	2 (6.1)		
	I bleed fewer days	11 (19.0)	3 (16.0)	8 (24.2)		
	My menstruation stops	4 (6.9)	0 (0)	4 (12.1)		

Abbreviations: LEAF-Q, Low Energy Availability in Females Questionnaire; LEA, low energy availability; NS, not significant.
Participants with LEAF-Q scores ≥8 were classified as being at risk of LEA.
